# Medial Epicondyle Fracture in Children and Its Association with Increased Carrying Angle

**DOI:** 10.1155/2022/6414247

**Published:** 2022-01-31

**Authors:** Changhoon Jeong, Sang-Uk Lee, Hyun Gyun Kim, Sun Young Joo

**Affiliations:** ^1^Department of Orthopedic Surgery, Bucheon St. Mary's Hospital, College of Medicine, The Catholic University of Korea, Seoul, Republic of Korea; ^2^Department of Orthopedic Surgery, Incheon St. Mary's Hospital, College of Medicine, The Catholic University of Korea, Seoul, Republic of Korea

## Abstract

**Background:**

This study aimed to evaluate the injury mechanism of medial epicondylar fractures in children and adolescents and its association with increased carrying angle (CA) as a predisposing factor.

**Materials and Methods:**

We evaluated 37 patients with medial epicondylar fractures who were surgically treated at our institution. Medical records and plain radiographs were reviewed to determine the mechanism of injury and the humerus-elbow-wrist angle (HEWA) and CA of the uninjured arm. To evaluate the effect of coronal alignment on specific fracture type, we compared the CA and HEWA of the 23 patients with medial epicondylar fracture who were injured by falling onto an outstretched hand (group I) with age- and sex-matched controls of 23 patients who had sustained extension-type supracondylar fractures (group II).

**Results:**

The mean age at injury was 11.7 ± 2.8 years (range, 5 to 16 years). Of the 37 patients, 23 (62.2%) recalled the injury mechanism as falling onto an outstretched hand and 10 patients (27.0%) were injured while arm wrestling, and in one patient (2.7%), the injury was associated with elbow dislocation. In the case-matched analysis, the mean HEWA of group I was 13.1 ± 2.8° (range, 7.1° to 19.8°) and the mean CA was 17.7 ± 2.7° (range, 13.0° to 22.2°). These angles were significantly increased in group I (*p*=0.003 and *p*=0.001, respectively).

**Conclusion:**

Falling onto an outstretched hand is the most common injury mechanism in patients with medial epicondylar fractures, and these fractures are associated with an increased CA.

## 1. Introduction

Medial epicondyle fractures are common, comprising 11% to 20% of all pediatric elbow fractures. These fractures mostly occur in boys between 9 and 14 years of age [1, 2]. It is known that medial epicondyle fractures are frequently associated with elbow dislocation, and the prevalence of elbow dislocation varies from 30% to 60% [1–4]. Bede et al., in their review of 50 patients with medial humeral epicondylar fractures, reported that 28 patients had concomitant elbow dislocation [3]. They concluded that an apparent isolated fracture of the medial humeral epicondyle is uncommon, and it is more frequently associated with elbow dislocation, with or without spontaneous reduction at the time of injury.

Previous studies on medial epicondyle fractures have mostly focused on the outcome of specific treatment methods, comparing the outcome of operative treatment with nonoperative management or treatment methods in patients with or without elbow dislocation [5, 6]. Historically, nonoperative treatment has shown favorable long-term outcomes even with fracture nonunion [7]. However, recently, there have been increasing concerns about symptomatic valgus instability in such nonunion fractures, and operative intervention is gaining popularity [8, 9]. Although the injury mechanism or a predisposing factor of this injury could influence the treatment strategy and clinical outcome, little is known about it.

Contrary to the study of Bede et al., we encountered more patients with isolated medial epicondylar fractures without dislocation who were injured by falling onto an outstretched hand. When a child falls onto an outstretched hand, elbow alignment can affect load transfer. Thus, theoretically the degree of angulation might associate with a specific type of elbow fracture. Therefore, in this study, we aimed to investigate the injury mechanism of medial epicondylar fractures in children and adolescents and its association with increased carrying angle (CA). We hypothesized that valgus alignment of the elbow is a predisposing factor for medial epicondylar fractures.

## 2. Materials and Methods

We performed a retrospective study after obtaining approval from our institutional review board. From 2015 to 2019, patients with medial epicondylar fractures surgically treated at our institution were included. Exclusion criteria were as follows: unknown injury mechanism, previous operation either on the injured arm or uninjured arm, or congenital or acquired deformity of either the injured or the contralateral uninjured upper extremity. We also excluded patients who did not undergo radiography of the uninjured elbow at the time of presentation. In total, 37 patients who met the inclusion criteria were included.

Patient demographics, such as age, sex, side of injury, treatment methods, and body mass index (BMI), were collected from the medical records. The injury mechanism was classified as follows: direct trauma, avulsion mechanism, and elbow dislocation. The avulsion mechanism was subdivided into two categories, injury during arm wrestling and falling onto the outstretched hand.

**Figure 1:**
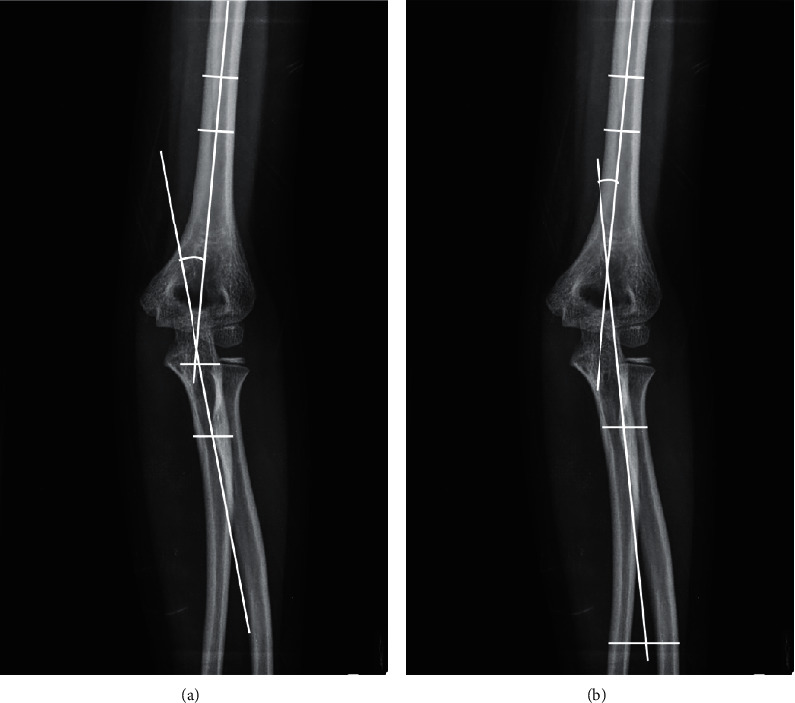
Radiographic measurements. The carrying angle (a) is the angle between the longitudinal axis of the humeral shaft and the longitudinal axis of the shaft of the ulna. The humerus-elbow-wrist angle (b) is the angle between the longitudinal axis of the humeral shaft and a line passing through the midpoints of 2 transverse lines across the forearm.

Anterior-posterior radiographs of the uninjured arm, taken at the time of presentation, were reviewed to evaluate the radiographic parameters. Radiographic measurements of the anterior-posterior radiographs of the elbow included the CA and the humerus-elbow-wrist angle (HEWA). The CA is the angle between the longitudinal axis of the humeral shaft and the longitudinal axis of the shaft of the ulna. The axis of the ulnar shaft was determined by a line passing through the midpoints of two transverse lines (1 proximal and 1 distal). The proximal line was drawn at the level of the olecranon, and the distal line was drawn at the level of the radial tuberosity. The HEWA is the angle between the longitudinal axis of the humeral shaft and a line passing through the midpoints of 2 transverse lines across the forearm. The proximal line was drawn at the level of the radial tuberosity, and the distal line was drawn at the level of the top of the radial bowing (Figure 1) [10].

All radiographs were independently reviewed by two orthopedic surgeons. The angle was measured at an interval of 2 weeks. We used the Picture Achieving and Communication System (PetaVision; Asan Medical Center, Seoul, Korea). Intraclass correlation coefficients (ICCs) were calculated according to standard statistical methods to assess the reliability of the measurements. Interobserver and intraobserver reliabilities of the measured radiologic parameters were assessed using correlation coefficients. ICCs were interpreted as follows: 0.00–0.20, poor; 0.21–0.40, fair; 0.41–0.60, moderate; 0.61–0.80, substantial; 0.81–1.00, perfect agreement. The radiographic measurements of the CA and HEWA showed good intra- and interobserver reliability, with ICCs ranging from 0.80 to 0.89.

To evaluate the effect of coronal alignment on specific fracture type, we compared the CA and HEWA of the 23 patients with medial epicondylar fracture who were injured by falling onto an outstretched hand (group I) with age- and sex-matched controls of 23 patients who had sustained extension-type supracondylar fractures (group II). Statistical analyses were performed using SPSS software (version 27; IBM Co., Armonk, NY, USA). Normal distribution was evaluated using the Kolmogorov–Smirnov test, and Student's *t*-test was performed for statistical analysis. A value of *p* < 0.05 was considered statistically significant.

## 3. Results

The mean age of the participants was 11.7 ± 2.8 years (range, 5.0 to 16.0 years) which included 28 boys (75.7%) and 9 girls (24.3%). In all, 25 patients (67.6%) had right-side involvement and 12 patients (32.4%) had left-side involvement. Operative treatment included open reduction and fixation with a 4.0 mm cannulated screw in 30 patients (81.1%) and open reduction and fixation with Kirschner wire in 7 patients (18.9%). Additional immobilization was performed in a long-arm cast for 4 weeks with the elbow flexed at 90°. The Kirschner wire was removed 4 weeks after surgery, and the cannulated screw was removed 6 months after surgery.

Avulsion was the most common injury mechanism, reported in 33 patients (89.2%). Specifically, fall onto an outstretched hand was encountered in 23 patients (62.2%), and avulsion during arm wrestling was observed in 10 patients (27.0%). Direct blow was observed in three patients (8.1%). Elbow dislocation was observed in one patient (2.7%). Injury during arm wrestling occurred in adolescents with the mean age of 14.0 ± 1.4 years (range, 12.0 to 16.0 years), whereas fall onto an outstretched hand occurred in the younger patients (mean age, 10.5 ± 2.7 years; range, 5.0 to 15.0 years). The mean CA was 18.1 ± 2.9° (range, 12.4° to 23.3°) and the mean HEWA was 13.1 ± 3.2° (range, 3.6 to 19.0°). The mean BMI was 19.4 ± 3.3 kg/m^2^ (range, 14.3 to 28.7 kg/m^2^; Table 1).

Table 2 shows the radiographic measurements of group I and group II. The mean HEWA of group I was 13.1 ± 2.8° (range, 7.1° to 19.8°) and the mean HEWA of the supracondylar fracture group was 10.0 ± 3.8° (range, 1.7 to 15.9°). The mean CA of group I was 17.7 ± 2.7° (range, 13.0° to 22.2°), whereas the CA in group II was 14.3° ± 3.6° (range, 3.6° to 19.5°). The mean HEWA and CA were significantly increased in group I (*p*=0.003 and *p*=0.001, respectively).

Furthermore, the mean BMI of group I was 19.6 ± 2.7 kg/m^2^ (range, 16.1 to 25.0 kg/m^2^) and was significantly greater than that of group II (17.6 ± 2.7 kg/m^2^; range, 14.4 to 25.2 kg/m^2^; *p*=0.037).

## 4. Discussion

**Figure 2:**
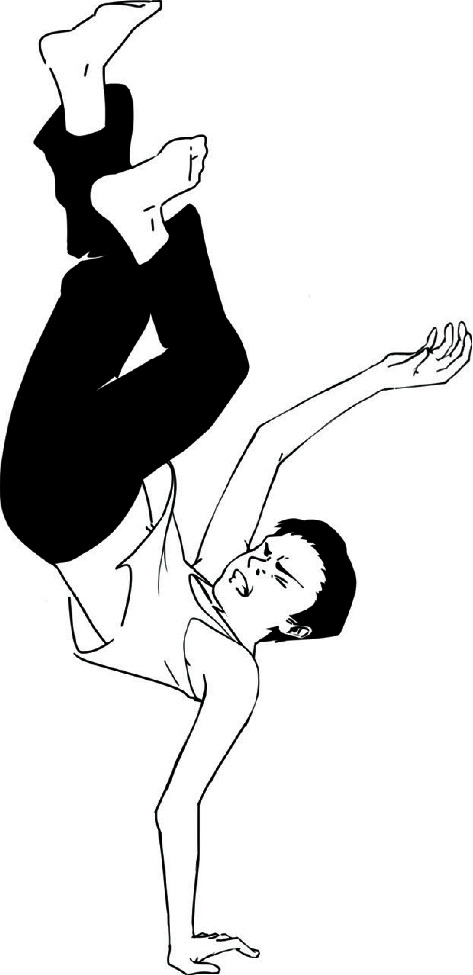
Injury mechanism of the fracture. Both medial epicondyle fracture and supracondylar fracture typically occur when a child falls onto an outstretched hand.

Three possible mechanisms of medial epicondylar fractures have been suggested [1, 11]: Direct blow, avulsion mechanism, and elbow dislocation. A direct blow on the posterior aspect of the medial epicondyle can cause fracture of the medial humeral epicondyle. In such injuries, the medial epicondylar fragment is often fragmented, and there may also be more superficial ecchymosis of the skin. The avulsion mechanism is more common and is caused by a forearm flexor-pronator muscular force. This muscle avulsion force can occur in combination with valgus stress in which the elbow is locked in extension, or as a pure muscular contraction that may occur with the elbow partially flexed. Avulsion during arm wrestling typically occurs due to sudden increased tension in the flexor-pronator group of muscles and subsequent avulsion of the medial humeral epicondyle [12–14]. Traction by violent contraction of flexors in the presence of mechanical imbalance is responsible for fracture-separation of the medial humeral epicondyle caused by arm wrestling [12]. Ogawa et al. suggested that injury occurs when patients were in a position that easily allowed them to move their body and only when one wrestler tried to force the end of the match and the other countered such a sudden move [12]. When the forearm flexors in a maximally contracted state are suddenly and passively stretched, a large traction force is applied to the medial humeral epicondyle resulting in an injury. This considerable traction force could be generated by the shift from concentric contraction of the flexors to eccentric contraction. Valgus stress on the elbow joint while falling on an outstretched hand is a common injury mechanism in the pediatric population (Figure 2). Smith proposed that when a child falls on his outstretched upper extremity with the elbow in extension, the wrist and fingers are often hyperextended, placing an added tension force on the epicondyle by the forearm flexor muscles [15]. The normal valgus CA tends to accentuate these avulsion forces when the elbow is in extension. The final proposed mechanism is associated with elbow dislocation, in which the ulnar collateral ligament provides the avulsion force. Dislocations of the elbow joint are rare in the first decade of life and become more common in older children involved in sporting activities, with a peak age of approximately 12 years [4, 16, 17]. The dislocation is most commonly posterolateral but may be posterior, lateral, or posteromedial. In 15 to 25% of elbow dislocations, the medial epicondyle is incarcerated in the joint [17]. Some patients may spontaneously relocate their elbows.

We investigated the prevalence of each injury mechanism causing medial epicondylar fractures in children. Our study showed a high prevalence of avulsion mechanism, as observed in 33 (89.2%) of our patients. Avulsion during arm wrestling was seen in adolescents and falling onto an outstretched hand was common in younger patients. Elbow dislocation was observed in only one patient; thus, it was uncommon in our group. This may be because vigorous sporting activities that lead to elbow dislocations are not popular among children and adolescents in Korea. Spontaneous reduction could be another explanation, as suggested by Bede et al. [3]. However, we excluded this possibility by evaluating the stability of the elbow under general anesthesia during surgery.

Although the degree of angulation or alignment affects the mechanism of load transfer, few studies have examined the association between elbow alignment and the specific type of fracture [18, 19]. Previous studies have focused on the effect of posttraumatic cubitus varus deformity on lateral condylar fracture [20–22]. Davids et al. reported six patients with lateral condylar fracture with preexisting cubitus varus deformity due to previous fracture malunion, and the biomechanical analysis suggests that both the torsional moment and the shear force generated across the capitellar physis by a routine fall are increased by varus malalignment [21]. Thus, posttraumatic cubitus varus may predispose a child to subsequent lateral condylar fractures. Similarly, Takahara et al. reported eight patients with lateral condylar fracture of the humerus after union of an ipsilateral supracondylar fracture that had healed with cubitus varus [2]. The study of David et al. supports that when the elbow is reinjured, due to a cubitus varus, the main force is varus.

Recently, a study by Kang and Park reviewed 374 patients with elbow fractures and compared the radiological CA of 34 pediatric patients with radial neck fracture with a case-matched comparison [23]. In their case-matched analysis, the CA of the patients with radial neck fracture was higher than that of patients with supracondylar fracture (14.3° vs 11.4°, *p*=0.013). Alternately, the CA was decreased in patients with lateral condylar as compared to the CA of patients with supracondylar fracture (7.7° vs 11.7°, *p* < 0.001). Their results suggest that elbow alignment could be a predisposing factor for specific types of pediatric elbow fractures.

**Figure 3:**
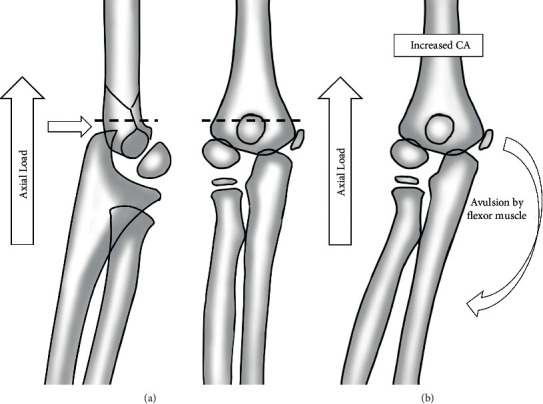
Illustration of load transmission when a child falls onto an outstretched hand. The elbow becomes locked into hyperextension. This converts the linear applied force to an anterior tension force. Posteriorly, the olecranon is forced into the depths of the olecranon fossa. As the bending force continues, the distal humerus fails anteriorly in the supracondylar area (a). In a valgus deviated elbow, the axial loading can be converted into avulsion force of the medial humeral epicondyle (b).

In the present study, we compared 23 patients with medial epicondyle fracture who were injured by falling onto an outstretched hand with 23 age- and sex-matched controls who had sustained a supracondylar fracture. We measured the radiographic CA instead of the clinical CA because it is more precise and reliable than the clinical measurement [24]. The HEWA has recently been adopted for the radiographic assessment of the coronal plane alignment of the elbow because the HEWA has good reliability and validity [10, 25]. The mean HEWA and CA were significantly increased in patients with medial epicondyle fracture compared to those with supracondylar fracture (17.6° ± 2.8° vs 14.3° ± 3.6°, *p*=0.001, and 13.1° ± 2.8° vs 10.0° ± 3.8°, *p*=0.003). This finding also suggested that increased valgus alignment in the elbow could be a predisposing factor for medial epicondyle fractures (Figure 3).

The mean BMI was higher in patients with medial epicondyle fracture compared to those with supracondylar fracture (19.6 ± 2.7 kg/m^2^ vs 17.6 ± 2.7 kg/m^2^, *p*=0.037). We think that when a child falls on his outstretched hand, in addition to the valgus alignment of the elbow, a higher body weight significantly increases the valgus force on the medial side of the elbow. The association with increased body weight and fracture risk in children is well understood [26]. However, it is unclear whether there is any relationship between increased body weight and specific type of fracture around the elbow.

However, our study had several limitations. First, due to the retrospective nature of this study, recall bias may have existed. Second, even though we compared the patients with age- and sex-matched controls, selection bias may be possible. Finally, we considered elbow alignment only in the coronal plane. Other factors that might affect load transmission, such as sagittal alignment, ligament laxity, and the position of the forearm (supination vs pronation), were not considered.

In conclusion, falling on an outstretched hand is the most common injury mechanism of medial epicondylar fractures in children and adolescents. Sustaining a medial epicondyle fracture during arm wrestling is also common among adolescents. Lastly, increased valgus alignment of the elbow was associated with medial epicondyle fractures.

## Figures and Tables

**Table 1:** Details of the patients according to the injury mechanism.

Variable	Direct blow	Avulsion		Elbow dislocation
		Avulsion during arm wrestling	Fall onto outstretched hand	
	*N* = 3	*N* = 10	*N* = 23	*N* = 1
Age (years)	14.0 ± 1.0	14.0 ± 1.4	10.5 ± 2.7	9.0
M: F	3 : 0	10 : 0	14 : 9	1 : 0
BMI (kg/m^2^)	17.4 ± 2.9	21.8 ± 4.9	19.6 ± 2.7	14.3
CA (°)	18.6 ± 3.2	18.5 ± 3.5	17.7 ± 2.7	22.2
HEWA (°)	12.2 ± 3.6	13.5 ± 4.4	13.1 ± 2.8	12.4

Values are presented as mean ± standard deviation. BMI, body mass index; CA, carrying angle; HEWA, humerus-elbow-wrist angle.

**Table 2:** Comparison of radiographic parameters with case-matched control.

Variable	Group I*∗* (*N* = 23)	Group II^†^ (*N* = 23)	*p* value
Age (years)	10.5 ± 2.7	10.0 ± 2.6	0.508
M: F	14 : 9	14 : 9	
R: L	16 : 7	6 : 17	
BMI (kg/m^2^)	19.6 ± 2.7	17.6 ± 2.7	0.037
CA (^o^)	17.7 ± 2.7	14.3 ± 3.6	0.001
HEWA (^o^)	13.1 ± 2.8	10.0 ± 3.8	0.003

Values are presented as mean ± standard deviation.*∗*Group I, medial epicondylar fracture; ^†^group II, supracondylar fracture; BMI, body mass index; CA, carrying angle; HEWA, humerus-elbow-wrist angle.

## Data Availability

The data that support the findings of this study are available on request from the corresponding author. The data are not publicly available due to privacy or ethical restrictions.
